# Extraocular Muscle Atrophy and Central Nervous System Involvement in Chronic Progressive External Ophthalmoplegia

**DOI:** 10.1371/journal.pone.0075048

**Published:** 2013-09-27

**Authors:** Cynthia Yu-Wai-Man, Fiona E. Smith, Michael J. Firbank, Grant Guthrie, Stuart Guthrie, Grainne S. Gorman, Robert W. Taylor, Douglass M. Turnbull, Philip G. Griffiths, Andrew M. Blamire, Patrick F. Chinnery, Patrick Yu-Wai-Man

**Affiliations:** 1 Department of Ophthalmology, Royal Victoria Infirmary, Newcastle upon Tyne, United Kingdom; 2 Institute of Cellular Medicine and Newcastle Magnetic Resonance Centre, Campus for Ageing and Vitality, Newcastle University, Newcastle upon Tyne, United Kingdom; 3 Institute for Ageing and Health, Campus for Ageing and Vitality, Newcastle University, Newcastle upon Tyne, United Kingdom; 4 Department of Neurology, Royal Victoria Infirmary, Newcastle upon Tyne, United Kingdom; 5 Wellcome Trust Centre for Mitochondrial Research, Institute for Ageing and Health, The Medical School, Newcastle University, Newcastle upon Tyne, United Kingdom; 6 Wellcome Trust Centre for Mitochondrial Research, Institute of Genetic Medicine, Newcastle University, Newcastle upon Tyne, United Kingdom; Centre for Eye Research Australia, Australia

## Abstract

**Background:**

Chronic progressive external ophthalmoplegia (CPEO) is a classical mitochondrial ocular disorder characterised by bilateral progressive ptosis and ophthalmoplegia. These ocular features can develop either in isolation or in association with other prominent neurological deficits (CPEO+). Molecularly, CPEO can be classified into two distinct genetic subgroups depending on whether patients harbour single, large-scale mitochondrial DNA (mtDNA) deletions or multiple mtDNA deletions secondary to a nuclear mutation disrupting mtDNA replication or repair. The aim of this magnetic resonance imaging (MRI) study was to investigate whether the ophthalmoplegia in CPEO is primarily myopathic in origin or whether there is evidence of contributory supranuclear pathway dysfunction.

**Methods:**

Ten age-matched normal controls and twenty patients with CPEO were recruited nine patients with single, large-scale mtDNA deletions and eleven patients with multiple mtDNA deletions secondary to mutations in *POLG*, *PEO1*, *OPA1*, and *RRM2B*. All subjects underwent a standardised brain and orbital MRI protocol, together with proton magnetic resonance spectroscopy in two voxels located within the parietal white matter and the brainstem.

**Results:**

There was evidence of significant extraocular muscle atrophy in patients with single or multiple mtDNA deletions compared with controls. There was no significant difference in metabolite concentrations between the patient and control groups in both the parietal white matter and brainstem voxels. Volumetric brain measurements revealed marked cortical and cerebellar atrophy among patients with CPEO+ phenotypes.

**Conclusion:**

The results of this study support a primary myopathic aetiology for the progressive limitation of eye movements that develops in CPEO.

## Introduction

Chronic progressive external ophthalmoplegia (CPEO) is a slowly progressive extraocular muscle disorder characterised by bilateral, usually symmetrical, limitation of eye movements and ptosis [1]. This classical manifestation of mitochondrial diseases can develop either in isolation or in association with other disabling neurological features, referred to as CPEO+ [2], [3]. Unsurprisingly, CPEO results in significant morbidity with a marked negative impact on the patient’s quality of life [4]. Molecularly, CPEO can be classified into two distinct genetic subgroups depending on whether patients harbour single, large-scale mitochondrial DNA (mtDNA) deletions or multiple mtDNA deletions secondary to a nuclear mutation disrupting mtDNA replication or repair [5], [6].

The aim of this magnetic resonance imaging (MRI) study was to investigate whether the limitation of eye movements in CPEO is myopathic in origin or whether there is evidence of contributory brainstem dysfunction with magnetic resonance spectroscopy (MRS). Brain volumetric measurements were also performed to assess the extent of central nervous system involvement and whether this correlated with the development of extraocular neurological features in patients with CPEO+ phenotypes.

## Patients and Methods

### Study Cohort

The study cohort included: (i) nine patients with single, large-scale mtDNA deletions (six females and three males, mean age = 50.8 years, standard deviation (SD) = 3.0 years), (ii) eleven patients with multiple mtDNA deletions (four females, seven males, mean age = 51.6 years, SD = 2.8 years), and (iii) ten age-matched normal controls (seven females and three males, mean age 50.3, SD = 2.3 years) (**Table 1**). Recruitment was limited to patients younger than 70 years of age to exclude possible age-related confounding factors, and to those with disease duration of more than five years. This study had the relevant institutional ethical approval (County Durham & Tees Valley 1 Research Ethics Committee, 08/H0905/106) and it was carried out in compliance with the Declaration of Helsinki. Participants provided their written consent for participation into this study.

**Table 1:** Clinical and molecular genetic characteristics of the CPEO cohort.

Patient	Genetic defect	Age	Sex	EOM limitation	Ptosis severity	Diplopia	Additional neurological complications
1	Single mtDNA deletion (2.9 Kb)	66	M	−3	Moderate	Yes	Myopathy, cerebellar dysfunction, migraine
2	Single mtDNA deletion (5.0 Kb)	45	F	−3	Severe	Yes	Myopathy, cerebellar dysfunction, fatigue
3	Single mtDNA deletion (7.7 Kb)	61	F	−4	Moderate	Yes	Myopathy
4	Single mtDNA deletion (5.0 Kb)	57	F	−3	Moderate	Yes	Myopathy
5	Single mtDNA deletion (6.5 Kb)	42	F	−3	Nil	Yes	Fatigue
6	Single mtDNA deletion (5.0 Kb)	54	M	−3	Severe	No	–
7	Single mtDNA deletion (4.8 Kb)	46	M	−4	Severe	No	–
8	Single mtDNA deletion (4.6 Kb)	41	F	−3	Moderate	Yes	–
9	Single mtDNA deletion (5.0 Kb)	45	M	−3	Severe	Yes	Myopathy, epilepsy
10	Multiple mtDNA deletions (*POLG*; p.A467T/p.Arg1096Cys)	42	M	−3	Severe	No	Myopathy, cerebellar dysfunction, peripheral neuropathy
11	Multiple mtDNA deletions (*POLG*; p.Trp748Ser/p.Arg1096Cys)	54	M	−2	Mild	Yes	Ataxia, epilepsy, peripheral neuropathy, cognitive impairment
12	Multiple mtDNA deletions (*POLG*; p.Ala467Thr/p.X1240Gln)	52	F	−3	Moderate	No	Ataxia, epilepsy, peripheral neuropathy
13	Multiple mtDNA deletions (*POLG*; p.Ala467Thr/p.Ala467Thr)	43	M	−3	Mild	Yes	Peripheral neuropathy, myalgia, cognitive impairment
14	Multiple mtDNA deletions (*PEO1*; p.Arg374Gln)	36	M	−3	Moderate	No	Myopathy, fatigue
15	Multiple mtDNA deletions (*PEO1*; p.Arg334Gln)	61	M	−3	Moderate	No	–
16	Multiple mtDNA deletions (*PEO1*; p.Leu381Pro)	58	F	−3	Severe	No	Myalgia
17	Multiple mtDNA deletions (*OPA1*; p.Ile432Val)	42	M	−2	Moderate	No	Myopathy, peripheral neuropathy, ataxia, cognitive impairment
18	Multiple mtDNA deletions (*RRM2B*; p.Ile224Ser)	61	F	−4	Moderate	Yes	Myopathy
19	Multiple mtDNA deletions (Unknown nuclear mutation)	60	M	−2	Severe	No	Myopathy, dysarthria
20	Multiple mtDNA deletions (Unknown nuclear mutation)	59	F	−3	Severe	No	Cerebellar dysfunction

EOM = extraocular muscle; F = female; M = male.

### Clinical and Molecular Investigations

All patients were assessed by an experienced multi-disciplinary team of neurologists and ophthalmologists to define the clinical phenotype and disease severity (**Table 1**). The limitation of eye movements was quantified from −1 to −4, with −1 indicating only mild limitation and −4 being the worst score, the eye being unable to move from the primary position of gaze [7]. Ptosis severity was graded according to the height of the palpebral aperture: (i) severe (<4 mm), (ii) moderate (4–6 mm), and (iii) mild (>6 mm) [8]. Additional histochemical and molecular investigations were carried out on skeletal muscle biopsies to confirm the clinical diagnosis of CPEO. A nuclear mutation was identified in nine of the eleven patients with CPEO found to harbour multiple mtDNA deletions: (i) *POLG* mutations (n = 4) [9], *PEO1* mutations (n = 3) [10], *OPA1* mutations (n = 1) [11], and *RRM2B* mutations (n = 1) [12]. For two patients, an underlying nuclear genetic defect has so far not been identified. Both these patients had typical clinical features of CPEO and mitochondrial histochemistry performed on skeletal muscle biopsies showed high levels of cytochrome *c* oxidase-deficient fibres in addition to prominent ragged red fibres.

### Magnetic Resonance Studies

MRI and MRS data were acquired on a 3-Tesla Philips Achieva clinical MR system using an 8-channel head coil. Proton MRS analysis was performed by an experienced research physicist (FES) to derive brain metabolite concentrations in the parietal white matter and brainstem regions: (i) choline; (ii) creatine; (iii) total glutamate and glutamine (Glx); (iv) myo-inositol; and (v) N-acetyl-aspartate (NAA). Extraocular and brain compartment volumes were measured using standardised semi-automated segmentation protocols (**Text S1**), with a high degree of intra- and inter-observer reliability (**Table S1**).

### Statistical Analysis

Statistical analysis was performed using GraphPad™ Prism v5 statistical software (San Diego, CA). Group comparisons were made using the unpaired t-test. Intra-observer and inter-observer variability for the measurement of extraocular muscle and brain volumes was assessed with Pearson correlation coefficient (r).

## Results

### Extraocular Muscle Morphology

**Figure 1:**
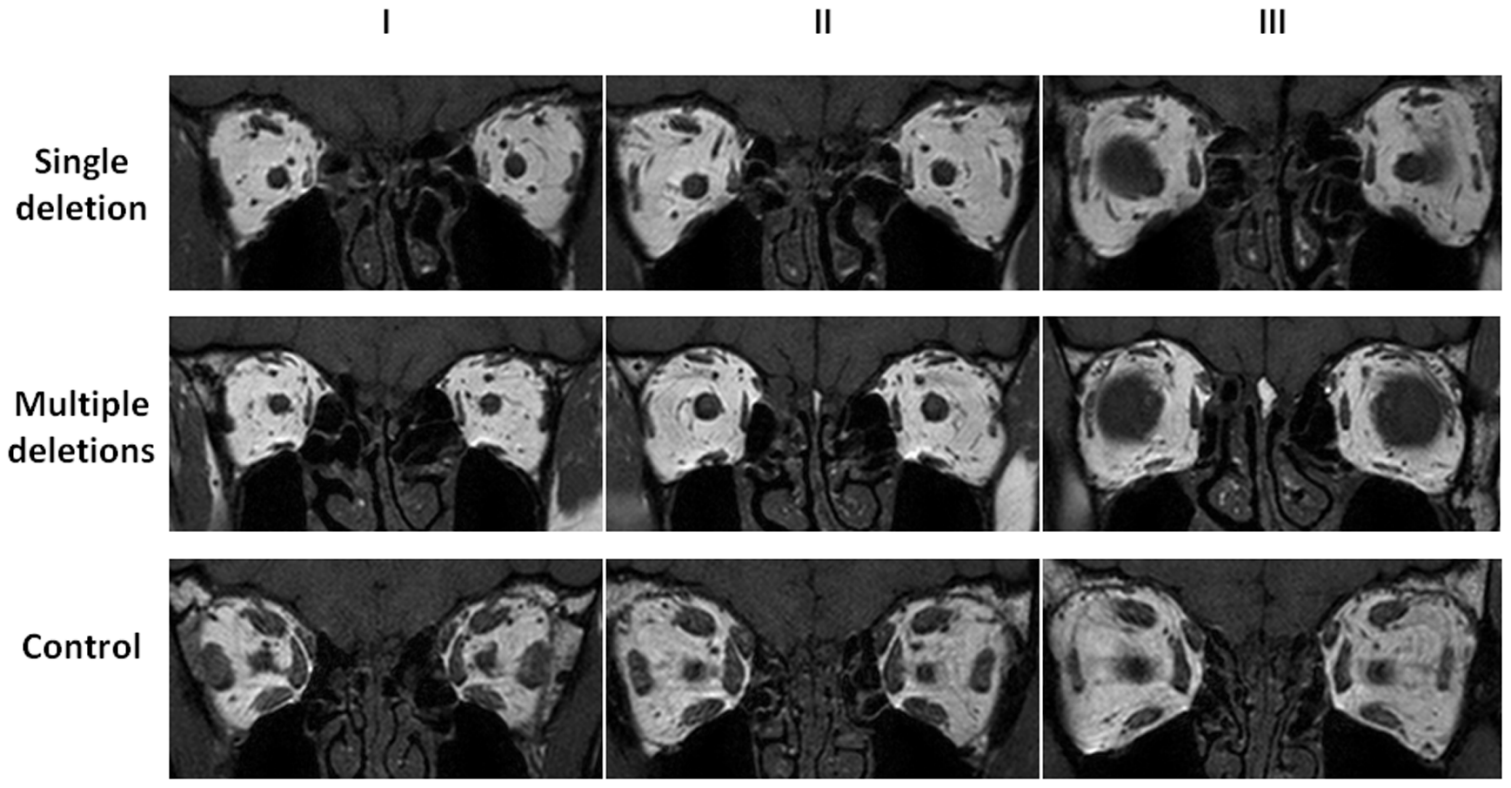
Extraocular muscle morphology in patients with CPEO and controls. Representative cross-sections of extraocular muscles have been provided at three different anatomical locations. All four recti muscles in patients harbouring single, large-scale deletions or multiple DNA deletions were atrophic compared with controls. Slice locations: I = 4 mm behind slice II towards the orbital apex; II = central slice; III = 4 mm in front of slice II towards the extraocular muscle insertions onto the globe.

**Figure 2:**
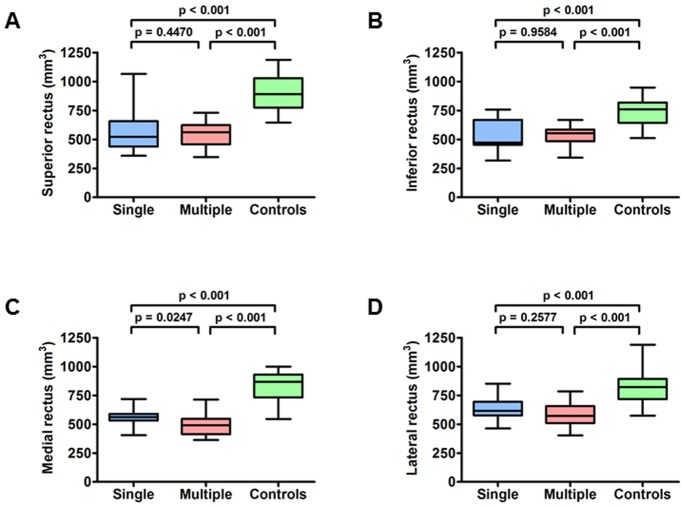
Comparison of extraocular muscle volumes between patients with CPEO and controls. Box plot of extraocular muscle volume data with the whiskers representing the minimum and maximum volumes. The ends of the boxes are the upper and lower quartiles, the vertical lengths of the boxes indicate the interquartile range, and the lines within the boxes represent the median volume for each group. CPEO cohort: single = single mtDNA deletion; multiple = multiple mtDNA deletions.

**Figure 3:**
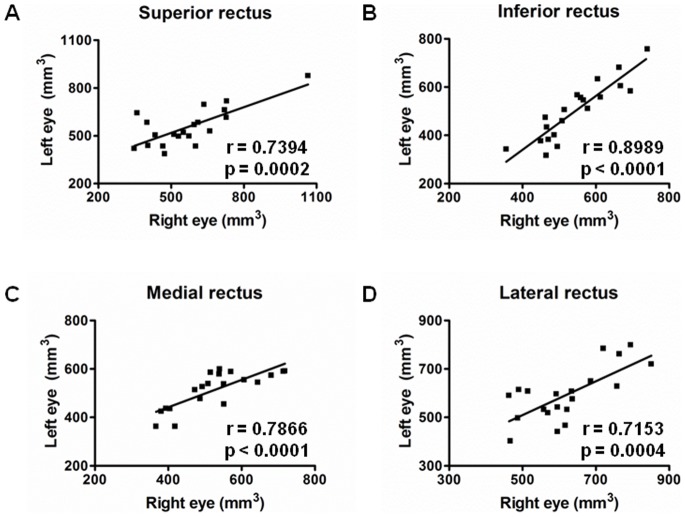
Interocular correlation of extraocular muscle volumes in patients with CPEO. r = Pearson correlation coefficient.

There was a significant reduction in extraocular muscle volumes in the CPEO group compared with normal controls for all four recti muscles (**Figure 1**), ranging from 24.7% to 40.2% (**Table 2** **and** **Figure 2**). No significant difference in extraocular muscle volumes was found between the two eyes from the same patient (**Table 3** **and** **Figure 3**). No specific MRI signal abnormalities were identified along the course of the extraocular muscles in patients with CPEO harbouring either single or multiple mtDNA deletions.

**Table 2:** Extraocular muscle volumes in patients with CPEO and controls.

Genetic group	Superior rectus	Inferior rectus	Medial rectus	Lateral rectus
	Mean ± SD (mm^3^) (%)^a^	Mean ± SD (mm^3^) (%)^a^	Mean ± SD (mm^3^) (%)^a^	Mean ± SD (mm^3^) (%)^a^
**Single mtDNA deletion** **(n = 9)**	582.6±41.8 (35.4%)	525.0±30.6 (28.2%)	556.6±17.9 (32.3%)	629.3±25.9 (24.7%)
	P<0.0001	P<0.0001	P<0.0001	P<0.0001
**Multiple mtDNA deletions** **(n = 11)**	547.3±23.7 (39.4%)	523.1±19.9 (28.5%)	491.8±20.3 (40.2%)	589.1±23.6 (29.5%)
	P<0.0001	P<0.0001	P<0.0001	P<0.0001
**Controls (n = 10)**	902.4±35.9	731.5±26.9	822.6±30.1	835.3±34.5

a Percentage reduction compared with controls. The respective P values indicate the level of significance for the comparisons with the control data set. SD: standard deviation.

**Table 3:** Interocular comparison of extraocular muscle volumes in patients with CPEO.

Anatomical side	Superior rectus	Inferior rectus	Medial rectus	Lateral rectus
	Mean ± SD (mm^3^)	Mean ± SD (mm^3^)	Mean ± SD (mm^3^)	Mean ± SD (mm^3^)
**Left eye (n = 20)**	556.4±27.0	502.5±26.8	514.0±17.2	593.6±24.6
**Right eye (n = 20)**	569.9±37.3	545.4±21.6	527.9±23.9	620.7±25.2
**P value**	0.7707	0.2205	0.6374	0.4469

The P value for each rectus muscle indicates the level of significance for the comparison between the left and right eye. SD = standard deviation.

### Brain Metabolites and Compartment Volumes

**Figure 4:**
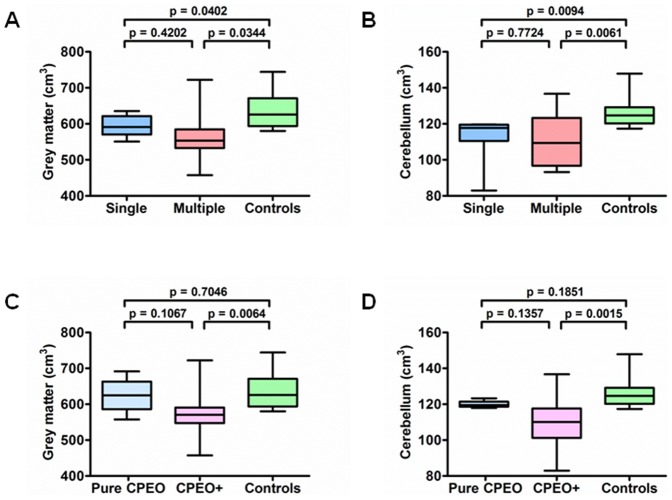
Comparison of brain compartment volumes between patients with CPEO and controls.

There was no significant difference in metabolite concentrations between the patient and control groups in both the parietal white matter and brainstem voxels (**Table 4**). There was a statistically significant reduction in total grey matter and cerebellar volumes for both patients with single deletions and multiple mtDNA deletions (**Table 5**). On subgroup analysis, the reduction in total grey matter and cerebellar volumes was apparent for patients with CPEO+, but not for those with pure CPEO phenotypes (**Figure 4**).

**Table 4:** Metabolite concentrations in parietal white matter and brainstem regions.

Voxel location	Subject group	Choline	Creatine	Glx	Myo-inositol	NAA
		Mean ± SD (mM)	Mean ± SD (mM)	Mean ± SD (mM)	Mean ± SD (mM)	Mean ± SD (mM)
**Parietal white matter**	**Single mtDNA deletion (n = 9)**	1.5±0.1	10.0±1.4	28.9±1.8	2.8±0.7	15.4±1.0
		P = 0.1970	P = 0.1428	P = 0.2636	P = 0.4628	P = 0.5933
	**Multiple mtDNA deletions (n = 11)**	1.4±0.1	10.4±0.5	25.6±1.6	1.8±0.3	13.0±0.9
		P = 0.7495	P = 0.0825	P = 0.0451	P = 0.1703	P = 0.0171
	**Controls (n = 10)**	1.3±0.3	12.5±2.7	32.8±2.9	4.4±2.1	16.0±0.7
**Brainstem**	**Single mtDNA deletion (n = 9)**	1.5±0.1	10.7±0.6	28.0±2.0	4.1±0.6	11.1±0.5
		P = 0.4266	P = 0.7425	P = 0.6837	P = 0.2336	P = 0.3711
	**Multiple mtDNA deletions (n = 11)**	1.4±0.1	10.0±1.2	23.5±3.1	4.2±0.9	11.8±0.9
		P = 0.3260	P = 0.5836	P = 0.4064	P = 0.2865	P = 0.8390
	**Controls (n = 10)**	1.7±0.6	11.0±2.5	26.7±2.2	5.9±1.3	12.1±0.8

The respective P values indicate the level of significance for the comparisons with the control data set, with P<0.0167 being the threshold level for statistical significance after Bonferroni correction for multiple testing.

**Table 5:** Volumetric brain measurements in patients with CPEO harbouring single and multiple mtDNA deletions.

Genetic group	Grey matter	White matter	Brainstem	Cerebellum
	Mean ± SD (cm^3^) (%)^a^	Mean ± SD (cm^3^) (%)^a^	Mean ± SD (cm^3^) (%)^a^	Mean ± SD (cm^3^) (%)^a^
**Single mtDNA deletion** **(n = 9)**	594.2±9.6 (6.6%)	437.9±14.2 (7.9%)	28.2±0.8 (7.2%)	112.6±3.9 (10.8%)
	P = 0.0402	P = 0.1168	P = 0.0575	P = 0.0094
**Multiple mtDNA deletions** **(n = 11)**	572.3±22.7 (10.1%)	462.8±20.7 (2.6%)	28.1±1.1 (7.6%)	111.0±4.0 (12.1%)
	P = 0.0344	P = 0.6516	P = 0.1012	P = 0.0061
**Controls (n = 10)**	636.5±15.8	475.3±17.3	30.4±0.6	126.3±2.7

a Percentage reduction compared with controls. The respective P values indicate the level of significance for the comparisons with the control data set. SD = standard deviation.

## Discussion

This study has shown clear evidence of significant extraocular muscle atrophy involving all four recti muscles in CPEO. This striking observation was noted for both patients with single, large-scale mtDNA deletions and for those harbouring multiple mtDNA deletions secondary to an underlying nuclear genetic defect. In a previous report of nine patients with CPEO, *Carlow and colleagues* observed a significant reduction in extraocular muscle volumes for the inferior rectus, medial rectus and lateral rectus [13]. The superior rectus was not assessed in their study and the diagnosis of CPEO was made on clinical grounds, precluding any genetic subgroup analysis. In a subsequent study, *Ortube and colleagues* measured extraocular muscle volumes in five patients with a clinical diagnosis of CPEO [14]. In contrast to the findings of our study, a reduction in extraocular muscle volume was noted for the superior rectus muscle only, but not for the other recti muscles despite a severe degree of ophthalmoplegia. There were, however, a number of limitations to the study by *Ortube and colleagues*, including the relatively small number of patients that were recruited and the lack of a confirmatory molecular diagnosis. Furthermore, only a relatively small portion of the extraocular muscle belly was sampled, which could have underestimated the degree of atrophy in the recti muscles.

Our study is the first to demonstrate objectively that the extent of extraocular muscle atrophy in CPEO is highly symmetrical between the two eyes of the same patient, reflecting the ophthalmoplegia pattern observed clinically [1]. No specific MRI signal abnormalities were identified throughout the whole length of the extraocular muscles in the twenty patients with CPEO that were investigated. A number of other ocular motility disorders can result in bilateral progressive ophthalmoplegia and differentiating these from CPEO can sometimes be challenging, especially when access to specialist mitochondrial genetic services is not routinely available. Although future prospective studies are needed to clarify this further, based on their characteristic extraocular muscle differences, MRI evaluation of the orbit could prove a useful adjunct in the diagnostic evaluation of this group of patients.

A variable decrease in N-acetyl-aspartate (NAA) has previously been reported in the brain of patients with the Kearns-Sayre syndrome – a particularly severe clinical phenotype characterised by the development of CPEO and pigmentary retinopathy before the age of twenty years, often in association with progressive cardiac conduction block [15], [16]. There was no significant difference in metabolite concentrations between the CPEO and control groups in our study. These proton MRS findings are consistent with the later onset and less severe neurological course in the patients that were recruited, none of whom fulfilled the criteria for the Kearns-Sayre syndrome.

Patients with CPEO+ features had significantly reduced total grey matter and cerebellar volumes compared with controls, in keeping with the higher burden of neurological disease in this specific patient population. The prominent degree of atrophy seen in the cerebellum further highlights the particular vulnerability of this specific brain region to the deleterious consequences of mtDNA defects [17], [18]. Patients with CPEO did not have significantly reduced brainstem volumes compared with controls and no brainstem metabolite abnormalities were detected with proton MRS. These observations, taken in conjunction with the marked atrophy of the extraocular muscles, support a primary myopathic origin for the progressive ophthalmoplegia seen in CPEO. However, some caution is required with regards to the subgroup comparisons given the relatively small number of patients in the single and multiple mtDNA deletion groups, and the different nuclear genetic defects involved. Furthermore, although CPEO is characterised by a significant myopathy involving the extraocular muscles, the degree of atrophy observed could still result, at least partly, from degeneration of the oculomotor nuclei as has been observed in some patients [19], [20].

This comprehensive magnetic resonance study of patients with molecularly confirmed CPEO has revealed a number of important findings that are directly relevant to our understanding of the underlying pathophysiology in this mitochondrial ocular disorder. In addition to marked generalised extraocular muscle atrophy, there was clear evidence of significant CNS involvement in this disorder, which becomes clinically manifest in a subgroup of patients.

## Supporting Information

Figure S1 **Voxel placements and proton magnetic resonance spectra.**
(PDF)

Figure S2 **Boundary delineation of extraocular muscle cross-sections.**
(PDF)

Figure S3 **Measurement protocol for brainstem and cerebellar volumes.**
(PDF)

Table S1 **Validation of extraocular muscle and brain volume measurements.**
(DOC)

Text S1 **Supplementary methods.**
(DOC)
