# Supplementary material for: Extraocular Muscle Atrophy and Central Nervous System Involvement in Chronic Progressive External Ophthalmoplegia
Source: PLoS One. 2013 Sep 27;8(9):e75048. doi: 10.1371/journal.pone.0075048 (PMC3785524; doi:10.1371/journal.pone.0075048)
Supplement: Figure S1 — Voxel placements and proton magnetic resonance spectra. (PDF) [file pone.0075048.s001.pdf]

Figure S1: Voxel placements and proton magnetic resonance spectra

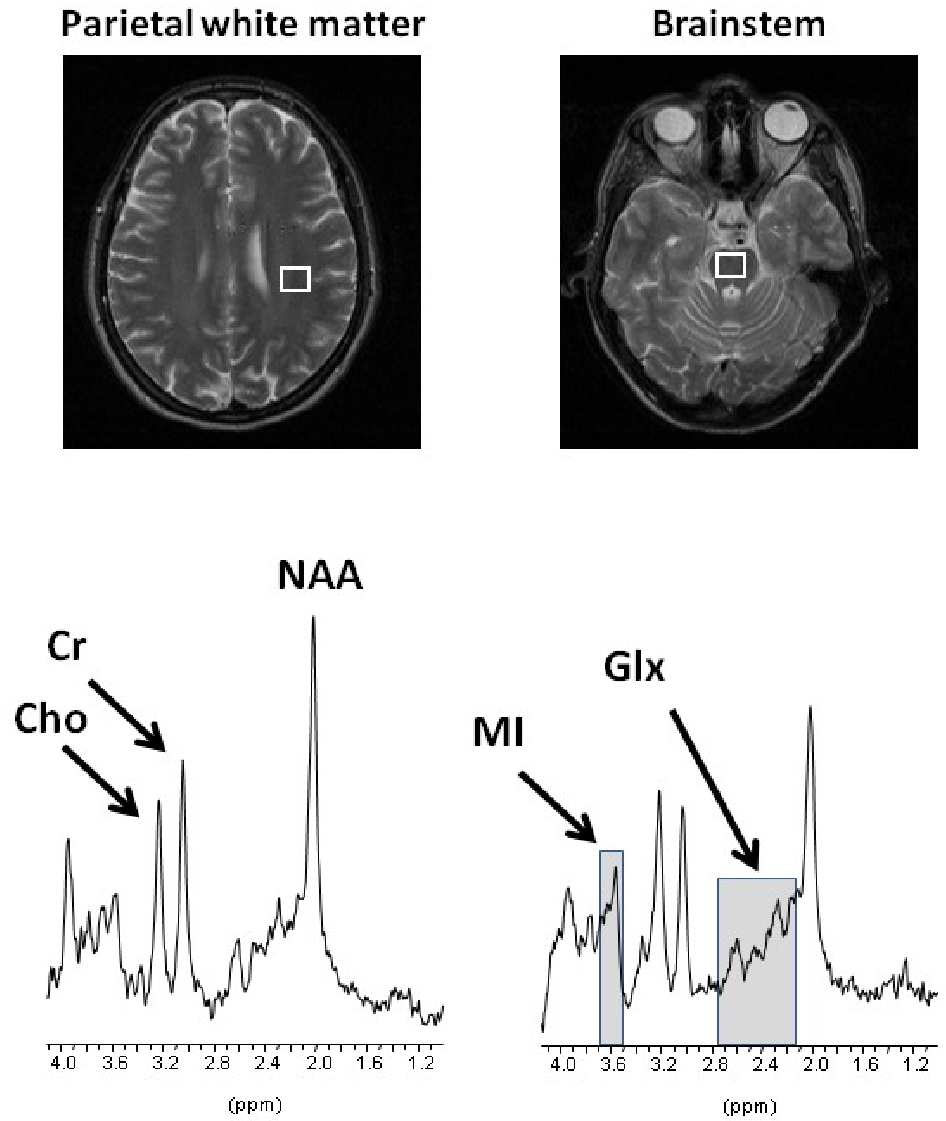

Axial T2-weighted MRI slices showing the voxel locations (white squares). The relevant metabolite peaks have been labelled in the lower panel: Cho = choline; Cr = creatine; Glx = total glutamate and glutamine; MI = myo-inositol; NAA = N-acetyl-aspartate.
