# Supplementary material for: Extraocular Muscle Atrophy and Central Nervous System Involvement in Chronic Progressive External Ophthalmoplegia
Source: PLoS One. 2013 Sep 27;8(9):e75048. doi: 10.1371/journal.pone.0075048 (PMC3785524; doi:10.1371/journal.pone.0075048)
Supplement: Figure S2 — Boundary delineation of extraocular muscle cross-sections. (PDF) [file pone.0075048.s002.pdf]

Figure S2: Boundary delineation of extraocular muscle cross-sections

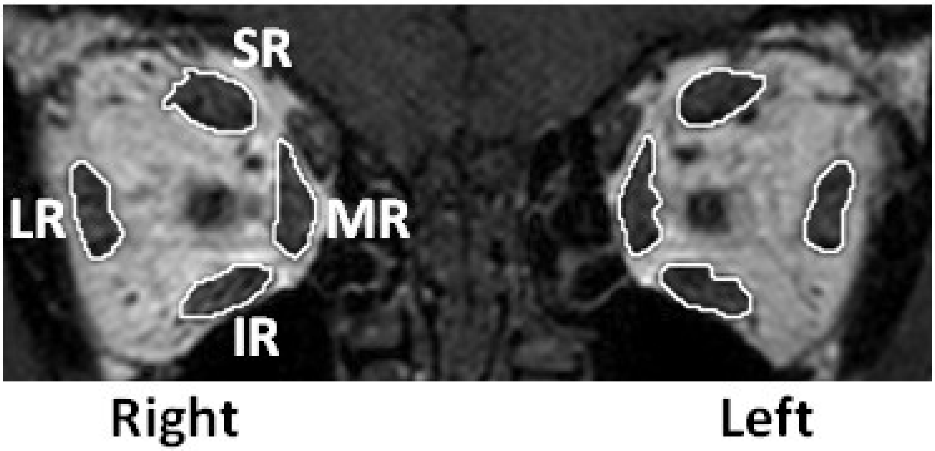

Coronal T1-weighted spin-echo MRI slice through the orbit with the boundaries of the recti muscles outlined. IR = inferior rectus; LR = lateral rectus; MR = medial rectus; SR = superior rectus.
