# Supplementary material for: Extraocular Muscle Atrophy and Central Nervous System Involvement in Chronic Progressive External Ophthalmoplegia
Source: PLoS One. 2013 Sep 27;8(9):e75048. doi: 10.1371/journal.pone.0075048 (PMC3785524; doi:10.1371/journal.pone.0075048)
Supplement: Figure S3 — Measurement protocol for brainstem and cerebellar volumes. (PDF) [file pone.0075048.s003.pdf]

Figure S3: Measurement protocol for brainstem and cerebellar volumes

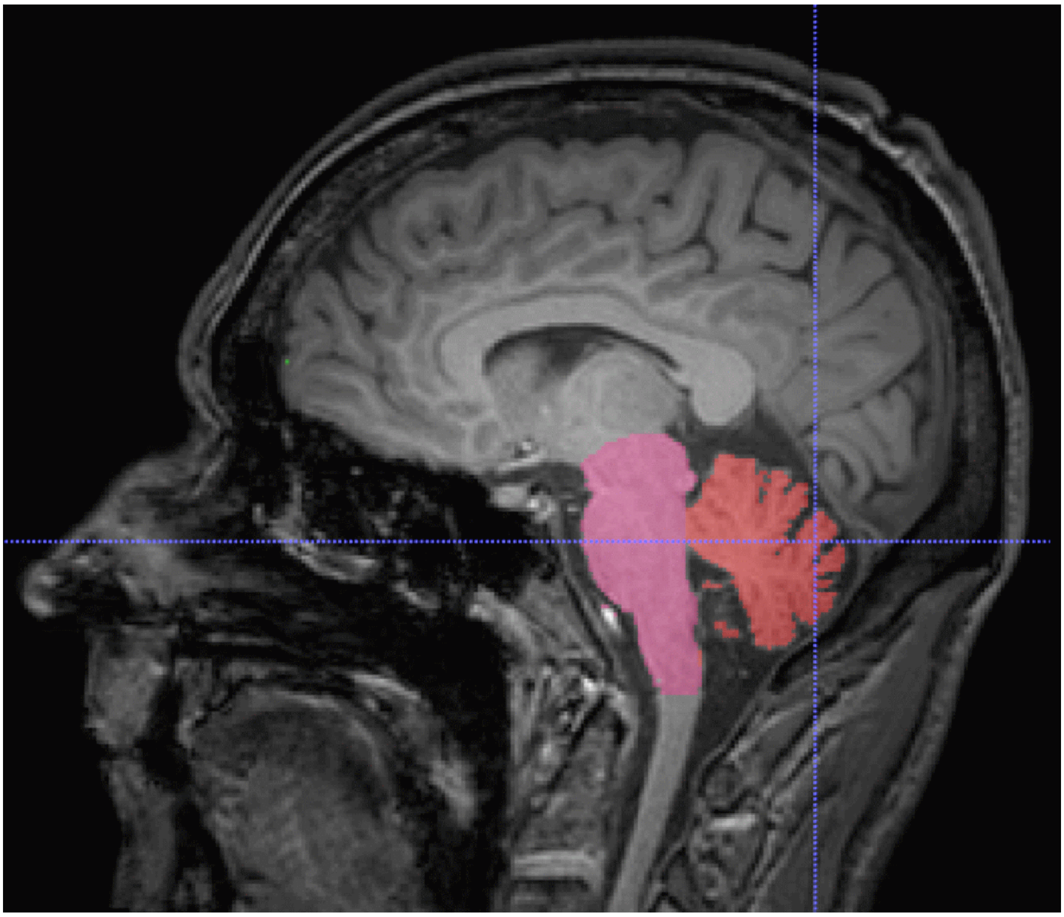

Sagittal T1-weighted MRI slice with the brainstem and cerebellar areas highlighted in pink and red, respectively.
