# Supplementary material for: Extraocular Muscle Atrophy and Central Nervous System Involvement in Chronic Progressive External Ophthalmoplegia
Source: PLoS One. 2013 Sep 27;8(9):e75048. doi: 10.1371/journal.pone.0075048 (PMC3785524; doi:10.1371/journal.pone.0075048)
Supplement: Table S1 — Validation of extraocular muscle and brain volume measurements. (DOC) [file pone.0075048.s004.doc]

**Table S1: Validation of extraocular muscle and brain volume measurements**

| **Volume measurements** | **Intra-observer correlation** | | **Inter-observer correlation** | |
| --- | --- | --- | --- | --- |
|  | **r =** | **P value** | **r =** | **P value** |
| **Superior rectus** | 0.9581 | < 0.0001 | 0.8766 | 0.0009 |
| **Inferior rectus** | 0.8898 | 0.0006 | 0.8148 | 0.0041 |
| **Medial rectus** | 0.9809 | < 0.0001 | 0.9102 | 0.0003 |
| **Lateral rectus** | 0.9008 | 0.0004 | 0.8127 | 0.0043 |
| **Brainstem** | 0.9941 | < 0.0001 | 0.9979 | < 0.0001 |
| **Cerebellum** | 0.9992 | < 0.0001 | 0.9988 | < 0.0001 |

r = Pearson correlation coefficient.
