# Supplementary material for: Extraocular Muscle Atrophy and Central Nervous System Involvement in Chronic Progressive External Ophthalmoplegia
Source: PLoS One. 2013 Sep 27;8(9):e75048. doi: 10.1371/journal.pone.0075048 (PMC3785524; doi:10.1371/journal.pone.0075048)
Supplement: Text S1 — Supplementary methods. (DOC) [file pone.0075048.s005.doc]

**Text S1**

**Supplementary Methods**

**Magnetic Resonance Studies**

MRI and MRS data were acquired on a 3-Tesla Philips Achieva clinical MR system using an 8-channel head coil. A conventional 3D T1-weighted volumetric MRI scan (MPRAGE, TR 8.3ms, TE 4.6ms, flip angle 8^o^, isotropic 1mm pixels) was initially performed to localise the regions of interest and for subsequent analysis of brain structure. To visualise the extraocular muscles, a coronal T1-weighted imaging sequence was then acquired perpendicular to the optic nerve plane: 24 slices of 1.5 mm thickness and 0.5 mm gap; TR 576 ms; TE 12 ms; pixel size 0.6 x 0.6 mm; field of view 180 x 180 mm. An anatomical T1-weighted magnetisation prepared rapid gradient echo (MPRAGE) scan was also generated covering the whole head (Sagittal acquisition; TR 9.6 ms; TE 4.6 ms; flip angle 8^o^; inversion delay 1250 ms; SENSE = 2; pixel size 1.15 x 1.15 x 1.2 mm; field of view 240 x 240 x 180 mm).

During each scanning session, all patients underwent a standardised proton MRS scanning protocol. Two voxels were sampled located within the parietal white matter (1.5 x 1.5 x 2.0 cm) and the brainstem (1.2 x 1.2 x 3.0 cm) regions (**Figure S1**), from which the following metabolites were quantified: choline, creatine, total glutamate and glutamine (Glx), myo-inositol, and N-acetyl-aspartate (NAA). Fully-relaxed MR spectra were acquired with a short TE PRESS sequence using a TR of 3 s and TE of 36 ms (128 averages, 1024 samples, spectral bandwidth 2000 Hz). A series of non-water suppressed spectra were also collected at 16 echo times ranging from 40 to 1500 ms with a TR of 10 s, from which the total tissue water was estimated and used as a reference for quantification of metabolite concentrations [1]. The total scan duration for both MRS acquisitions was 8.5 minutes.

**Derivation of Brain Metabolite Concentrations**

Proton MRS analysis was performed by an experienced research physicist (FES) using the automated standard evaluation software jMRUI [2]. Residual water peaks were filtered with a Hankel Lanczos Singular Values Decomposition Filter (HLSVD) [3]. MRS data was then evaluated in the time domain with the QUEST algorithm, which fitted a weighted combination of metabolite signals directly to the *in vivo* spectroscopic data. The algorithm used prior knowledge based on calibrations of known metabolite concentrations measured *in vitro*. Absolute concentrations of brain metabolites were determined against the internal water signal collected from the same recording voxel as the metabolite data. Discrimination between cerebrospinal fluid (CSF) and tissue water in the sampled voxel was determined by extrapolation of the fitted double exponential decay arising from the differences in the T2 relaxation times of CSF and tissue water, using a least squares fitting algorithm written in Matlab (Mathworks Inc., Massachusetts, USA) [1].

**Quantification of Extraocular Muscle Volumes**

Extraocular muscle volumes were analysed using a semi-automated segmentation technique on the five central slices of the muscles [4,5]. Extraocular muscle measurements for the whole study group were performed by a single observer (CYWM) who was blinded to the subject’s identity. Regions of interest were drawn around the superior rectus, inferior rectus, medial rectus and lateral rectus on each slice, using intensity thresholding to identify the fat-muscle boundary and manual tracing for the other boundaries (**Figure S2**). Intra-observer (CYWM) and inter-observer reliability (CYWM and MJF) for the measurement of extraocular muscle volumes was carried out on five randomly selected subjects, with 10 muscles each for the superior rectus, inferior rectus, medial rectus and lateral rectus groups. The measurement protocol used had a high degree of intra- and inter-observer reliability (**Table S1**).

**Volumetric Brain Compartment Analysis**

The generated brain MPRAGE scans were segmented into grey and white matter and CSF using the unified segmentation in SPM8 ([www.fil.ion.ucl.ac.uk/spm](http://www.fil.ion.ucl.ac.uk/spm)) [6,7]. The cerebellum and brainstem were segmented by transforming regions of interest from the Montreal Neurological Institute (MNI) space for each individual brain (**Figure S3**). The cerebellum regions of interest were taken from the MNI probabilistic template distributed as part of FSL (<http://www.fmrib.ox.ac.uk/fsl/>). The superior boundary of the brainstem was set to a line connecting the anterior and posterior commissures. The inferior boundary of the brainstem was set at the decussation of the pyramids and foramen magnum. The lateral boundaries of the brainstem were the point of separation of the superior and middle cerebellar peduncles. The cerebellum and brainstem regions of interest were then transformed using the spatial normalisation calculated in the SPM segmentation into the native space of each subject’s brain, allowing the cerebellum and brainstem volumes to be generated for that subject. These segmentations were then manually edited, where necessary, using the ITK software ([www.itksnap.org](http://www.itksnap.org/)). Brain volume measurements for the whole study group were performed by a single observer (GG) who was blinded to the subject’s identity. Intra-observer (GG) and inter-observer reliability (GG and SG) for the measurement of brainstem and cerebellar volumes was carried out on seven randomly selected subjects. There was a high degree of intra- and inter-observer reliability in the measurement of brainstem and cerebellar volumes (**Table S1**).

**Supplementary References**

1. Ernst T, Kreis R, Ross B (1993) Absolute quantification of water and metabolites in the human brain. I. Compartments and water. J Magn Reson Series B 102: 1-8.

2. Stefan D, Di Cesare F, Andrasescu A, Popa E, Lazariev A, Vescovo E (2009) Quantitation of magnetic resonance spectroscopy signals: the jMRUI software package. Meas Sci Tech 20: 104035: 1-9.

3. Pijnappel WWF, Vandenboogaart A, Debeer R, Vanormondt D (1992) SVD-based quantification of magnetic-resonance signals. J Magn Reson 97: 122-134.

4. Firbank MJ, Coulthard A (2000) Evaluation of a technique for estimation of extraocular muscle volume using 2D MRI. Br J Radiol 73: 1282-1289.

5. Firbank MJ, Harrison RM, Williams ED, Coulthard A (2001) Measuring extraocular muscle volume using dynamic contours. Magn Reson Imaging 19: 257-265.

6. Ashburner J, Friston KJ (2005) Unified segmentation. Neuroimage 26: 839-851.

7. Good CD, Johnsrude IS, Ashburner J, Henson RN, Friston KJ, Frackowiak RS (2001) A voxel-based morphometric study of ageing in 465 normal adult human brains. Neuroimage 14: 21-36.
